# The developmental biogeography of hawksbill sea turtles in the North Pacific

**DOI:** 10.1002/ece3.2034

**Published:** 2016-03-08

**Authors:** Kyle S. Van Houtan, Devon L. Francke, Sarah Alessi, T. Todd Jones, Summer L. Martin, Lauren Kurpita, Cheryl S. King, Robin W. Baird

**Affiliations:** ^1^ NOAA FisheriesPacific Islands Fisheries Science CenterHonoluluHawaii96818; ^2^ Nicholas School of the EnvironmentDuke UniversityDurhamNorth Carolina27708; ^3^ Joint Institute for Marine and Atmospheric ResearchUniversity of HawaiiHonoluluHawaii96822; ^4^ National Research CouncilNational Academy of SciencesWashingtonDistrict of Columbia20001; ^5^ Pacific Cooperative Studies UnitUniversity of HawaiiHonoluluHawaii96822; ^6^ World Turtle TrustKailuaHawaii96734; ^7^ Hawaii Wildlife FundPaiaHawaii96779; ^8^ Cascadia Research CollectiveOlympiaWashington98501; ^9^ Present address: Monterey Bay AquariumMontereyCalifornia93940

**Keywords:** Bycatch, coastal ecology, natal dispersal, ocean plastics, pollution, spatial structure

## Abstract

High seas oceanic ecosystems are considered important habitat for juvenile sea turtles, yet much remains cryptic about this important life‐history period. Recent progress on climate and fishery impacts in these so‐called lost years is promising, but the developmental biogeography of hawksbill sea turtles (*Eretmochelys imbricata*) has not been widely described in the Pacific Ocean. This knowledge gap limits the effectiveness of conservation management for this globally endangered species. We address this with 30 years of stranding observations, 20 years of bycatch records, and recent simulations of natal dispersal trajectories in the Hawaiian Archipelago. We synthesize the analyses of these data in the context of direct empirical observations, anecdotal sightings, and historical commercial harvests from the insular Pacific. We find hawksbills 0–4 years of age, measuring 8–34 cm straight carapace length, are found predominantly in the coastal pelagic waters of Hawaii. Unlike other species, we find no direct evidence of a prolonged presence in oceanic habitats, yet satellite tracks of passive drifters (simulating natal dispersal) and our small sample sizes suggest that an oceanic phase for hawksbills cannot be dismissed. Importantly, despite over 600 million hooks deployed and nearly 6000 turtle interactions, longline fisheries have never recorded a single hawksbill take. We address whether the patterns we observe are due to population size and gear selectivity. Although most sea turtle species demonstrate clear patterns of oceanic development, hawksbills in the North Pacific may by contrast occupy a variety of ecosystems including coastal pelagic waters and shallow reefs in remote atolls. This focuses attention on hazards in these ecosystems – entanglement and ingestion of marine debris – and perhaps away from longline bycatch and decadal climate regimes that affect sea turtle development in oceanic regions.

## Introduction

Compared to other species of sea turtles, the early life history of hawksbills is relatively undescribed. Sea turtles display some biogeographic and life‐history variability (Bolten 2003), but all species – with the exception of the flatback (*Natator depressus*) – are thought to have a significant juvenile oceanic development phase (Polovina et al. 2004; Reich et al. 2007; Shillinger et al. 2012). While it is known that juvenile and adult hawksbill sea turtles inhabit coral reefs, mangrove estuaries, and other hard‐bottom habitats (Meylan 1988; Bjorndal and Bolten 2010; Gaos et al. 2012a), little evidence exists documenting the first years of development (e.g., Putman et al. 2014). Resolving this gap in the spatial population structure of hawksbills is important for understanding how anthropogenic and climate indices together impact the dynamics and conservation status of the species (Van Houtan and Halley 2011; Van Houtan et al. 2015).

For centuries, hawksbills were heavily exploited in the global tortoiseshell trade, threatening all populations with extinction (Groombridge and Luxmore 1989). Recent population trends are more encouraging (e.g., Richardson et al. 2006; Beggs et al. 2007; Hamilton et al. 2015), yet hawksbills remain endangered under the U.S. Endangered Species Act and the IUCN Red List considers them critically endangered (Mortimer and Donnelly 2008; NMFS and USFWS 2013) – both listings being the most imperiled status for extant species. In Hawaii, the hawksbill population is particularly isolated and small, with roughly 100 breeding females in existence (Hutchinson et al. 2008; Seitz et al. 2012). This means Hawaii hawksbills are perhaps the smallest distinct sea turtle population on the planet (Van Houtan et al. 2012, 2016), and therefore, their population dynamics and conservation threats deserve urgent attention.

To better understand the developmental biogeography of hawksbills, we present new analyses of strandings and necropsies (including gut contents), satellite drifters simulating hatchling dispersal, longline bycatch, and anecdotal sightings – all from the Hawaiian archipelago and surrounding waters in the central North Pacific Ocean. Detailed dietary studies are often essential for understanding wildlife behavior, habitat, and population threats (Sekercioglu et al. 2002) particularly for sea turtles (Parker et al. 2005, 2011; Boyle and Limpus 2008; Schuyler et al. 2014; Seminoff et al. 2014; Van Houtan et al. 2014; Santos et al. 2015). Commercial fisheries bycatch (Lewison et al. 2004; Peckham et al. 2007; Finkbeiner et al. 2011; Roe et al. 2014) and stranding (Epperly et al. 1996; Chaloupka et al. 2008; Van Houtan et al. 2010) data also shed light on habitat association and foraging ecology. More recently, ingestion of anthropogenic debris has become an increasing concern for marine species (Schuyler et al. 2014; Vegter et al. 2014) as it is a direct source of mortality, a vector for persistent organic pollutants, and thought to delay sea turtle development particularly for pelagic life stages (Santos et al. 2015).

We focus attention on hawksbills ranging from 8 to 34 cm in straight carapace length (hereafter “length”), a seldomly observed developmental stage in the Pacific Ocean and in other populations (Grant et al. 1997). As a result, we report new insights on the habitat of turtles in this population segment with potential application to other ocean regions. In addition, we present novel summaries of sea turtle bycatch for the Hawaii longline fisheries and discuss the threat of ingested anthropogenic debris. Our aim is part of our larger project to bring diverse data streams to bear on complex and data‐poor conservation challenges.

## Methods

### Strandings and necropsies

Stranding records were gathered by NOAA's Pacific Islands Fisheries Science Center, Protected Species Division, from July 1982 to March 2015. These data include morphometrics, location, disposition, cause of stranding, and (for dead turtles) necropsy findings for all Hawaii sea turtle strandings (e.g., Chaloupka et al. 2008; Van Houtan et al. 2010). Separate, in‐depth analyses of population threats revealed from strandings (Brunson et al. in review) and foraging habits (T. T. Jones, K. S. Van Houtan, and C. S. King in prep) are forthcoming.

We calculated the straight distance to shore for each hawksbill stranding using the responder narrative and/or recorded GPS location of each event. Size classes are based on published categories (Seitz et al. 2012; Van Houtan et al. 2016) and are further binned within these categories to maximize data resolution while distributing samples evenly (≥10 turtles) across bins. As a point of reference, we calculate the 95% interval from the normal distribution of distances from all bins except the cryptic 8–34 cm length class.

For the single posthatchling available for necropsy, we examined the entire gastrointestinal tract and collected its contents. We air‐dried the dietary items on a tray in a 5°C walk‐in refrigerator for 48 h, separated the items into three broad categories of items present – algae, plastic, and beetles – and stored in heavyweight poly bags (ULINE^™^, Pleasant Prairie, Wisconsin, USA recloseable 4 mil) with SiO_2_ indicating gel desiccant (Fisher Scientific^™^ Waltham, Massachusetts, USA grade 48, 4–10 mesh). Once completely dried, we recorded the total mass of each group using an analytical balance readable to 0.0001 g (Acculab^™^ ALC‐210.4 Sartorius AG, Göttingen, Germany). After weighing, we stored each group in graduated 1.5 mL cryovials (Thermo Fisher Scientific^™^, Waltham, Massachusetts, USA Nalgene^™^), recording the dry volume. For algae, we converted dry to wet volume assuming 86.5% water content (Angell et al. 2012). For plastic debris, we counted the total pieces, recorded their color, and measured their length and width dimensions (Bugoni et al. 2001; Mascarenhas et al. 2004; Lazar and Gračan 2011) with a Greenough stereomicroscope (scope: Leica^™^ S8 APO, camera: Leica^™^ DFC295, firmware: Leica^™^ Microsystems, Buffalo Grove, Illinois, USA Applications Suite). We consulted the entomology staff at the Bernice Pauahi Bishop Museum to identify the beetles.

To age the posthatchling, we obtained the dates of observed hatchling emergences from Hawaii and Maui (the primary nesting grounds, e.g., Seitz et al. 2012; Van Houtan et al. 2012) for the 2014 nesting season. We calculated the first, second, and third quartile (median, 50% confidence interval) of the empirical distribution of emergence dates and subtracted these values from the stranding date thus providing a low, median, and high age estimate.

### Hatchling dispersal trajectories

Surface drifters are solar‐powered pop‐up satellite archival (PSAT) tags (Desert Star Systems^™^, Marina, California, USA SeaTag‐GEO) equipped with temperature and light sensors, and Argos location. Each cylindrical tag weighs 45 g and measures 14 × 112 mm (including a syntactic foam float, with an additional 1 × 140 mm antenna). Drifters were released in the ocean at two sites to record passive drift trajectories that might parallel conditions for green and hawksbill posthatchling turtles in the Hawaiian archipelago. For hawksbills, we released two drifters off Ewa Beach, Oahu (21.296°N, 157.975°W) in December 2013. For green turtles, as a comparison, we released four drifters at East Island, French Frigate Shoals (FFS), NWHI (23.788°N, 166.209°W) in July–August 2014. The FFS drifters match the exact time and location of peak green turtle hatchling emergences in Hawaii. Due to logistical constraints, the hawksbill‐mimicking (OSS) drifters were slightly modified from ideal conditions (i.e., a month past peak hatchling emergence, not on Hawaii Island). Given the observed prevailing currents in the Hawaiian Archipelago (Howell et al. 2012; Carson et al. 2013); however, either method would likely generate similar results.

We built drifter trajectory paths with the transmitted coordinates from each PSAT tag using only locations with Argos quality codes 3, 2, 1, 0, A, and B (excluding Z). Using all transmissions from tag deployment to March 2015, we assessed whether the tag was still active and calculated the total transmission time and tag life. We mapped the trajectories and relevant landmarks using ArcGIS 9.3 (ESRI 2009).

### Fishery bycatch

Bycatch data from the Hawaii‐based longline fishing fleets are from NOAA's Pacific Islands Regional Office, Observer Program from 1994 to 2014, and from summary reports (McCracken 2000; Boggs et al. 2009). NOAA observers on fishing vessels recorded the location, species, and length of bycaught turtles. Turtle bycatch is from two longline fleets – shallow‐set and deep‐set – that operate in different geographic regions, target different fish (swordfish and tunas, respectively), and deploy different gear rigs at different depths (Bartram and Kaneko 2004). Due to partial observer coverage (approximately 20%) in the deep‐set fleet, total turtle bycatch is estimated (McCracken 2000). Set locations of the longline fisheries 1994–2015 were provided by NOAA's Pacific Islands Fisheries Science Center, Fisheries Monitoring Branch.

We tabulated the total observed annual sea turtle bycatch in the Hawaii‐based longline fisheries from 1994 to 2014 and plotted its spatial distribution and length composition by species. As we are interested in the location of developmental stages of sea turtles, we averaged the length of turtles caught in each 5° × 5° area (to maintain fishery operator confidentiality) for each species. We also generated length frequency plots of turtle demographics for the fisheries. Occasionally, lengths are calculated from observer sight estimates of total turtle length. For spatial context, we calculated and plotted the 95% kernel volume contour of the geographic extent of each longline fishery since 1994.

## Results

**Figure 1:**
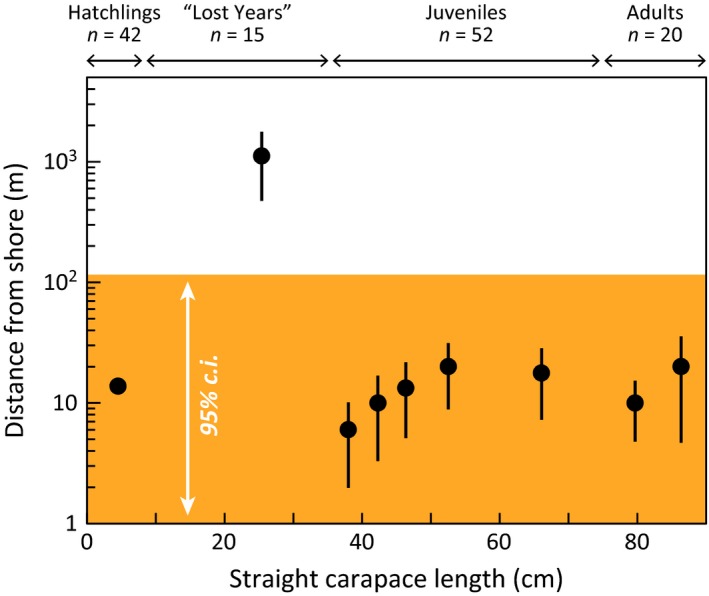
Proximity of hawksbill strandings to the Hawaii coastline across all life stages. Most size classes strand within 100 m of the low tide line in nearshore neritic habitat. The lone exception is turtles in the 8–34 cm class that are commonly observed >1 km from the coast. Black circles are average class distance, bars are standard error, and orange region is the 95% confidence interval for all groups, save the 8–34 cm turtles. For each size class, n ≥ 10, representing 129 hawksbill strandings from 1984 to 2015.

Figure 1 plots the distance stranded from shore for 128 Hawaii hawksbills throughout ontogeny. Hatchlings (3.0–7.9 cm, *n *=* *42), juveniles (35.0–74.4 cm, *n *=* *52), and adults (74.5–90.0 cm, *n *=* *20) are nearshore, stranding within 30 m of the coast. The cryptic stage (8.0–34.9 cm, *n *=* *15), however, has been documented stranding offshore in pelagic waters proximate the archipelago, and this particular data point and its entire error interval is outside the confidence interval from the other size classes. Three turtles documented >1 km offshore, in particular, measured 13, 27, and 31 cm and were all released from fishing gear entanglements near Hawaii, Maui, and Molokai islands, respectively. One additional turtle excluded from this analysis (due to time and therefore distance drifted since death) was a highly decomposed 30.7 cm juvenile recovered from a derelict fishing net at sea (21.981°N, 167.000°W) roughly 200 km SSW from FFS in the NWHI.

**Figure 2:**
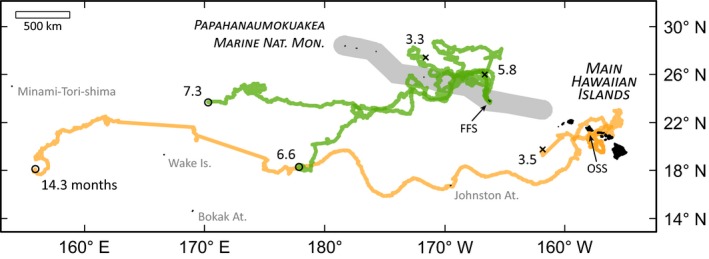
Surface drifter trajectories from hawksbill and green turtle nesting areas in the Hawaiian Archipelago indicate young juveniles may reside near the archipelago for several months or more. Green lines are 4 PSAT drifters released from French Frigate Shoals (FFS) in July–August 2014, simulating green turtle posthatchling trajectories from their primary nesting beach in the northwestern Hawaiian Islands. Orange lines are 2 PSAT surface drifters released near Oahu's south shore (OSS) in December 2013, simulating hawksbill posthatchling trajectories from the Main Hawaiian Islands. The timing and location of release parallel predominant conditions for both populations. Paths are Argos location codes 3‐B, “x” at path endpoint indicates transmission ends, “o” indicates drifter still active, and number is trajectory age in months. Gray region is the extent of the Papahanaumokuakea Marine National Monument.

Passive drifters released in the vicinity of the primary green and hawksbill nesting grounds in the Hawaiian Islands had variable trajectories with no single path (Fig. 2). For both sets of releases, drifters took meandering paths proximate (<200 km) to the archipelago in pelagic waters for several or more months. Three of the drifters stopped transmitting; two released at FFS (3.3 and 5.8 months); and one released off Oahu (3.5 months). These three drifters all remained <300 km from land. All drifters with a lifetime of >6 months, however, moved westerly into oceanic waters following the North Equatorial Current across the dateline (180°). The longest transmitting drifter (>14 months) was released off Oahu, drifted through Johnston Atoll, passed north of Wake Island, and was last observed at 18.092°N, 155.904°E, roughly 4800 km from its release site.

**Figure 3:**
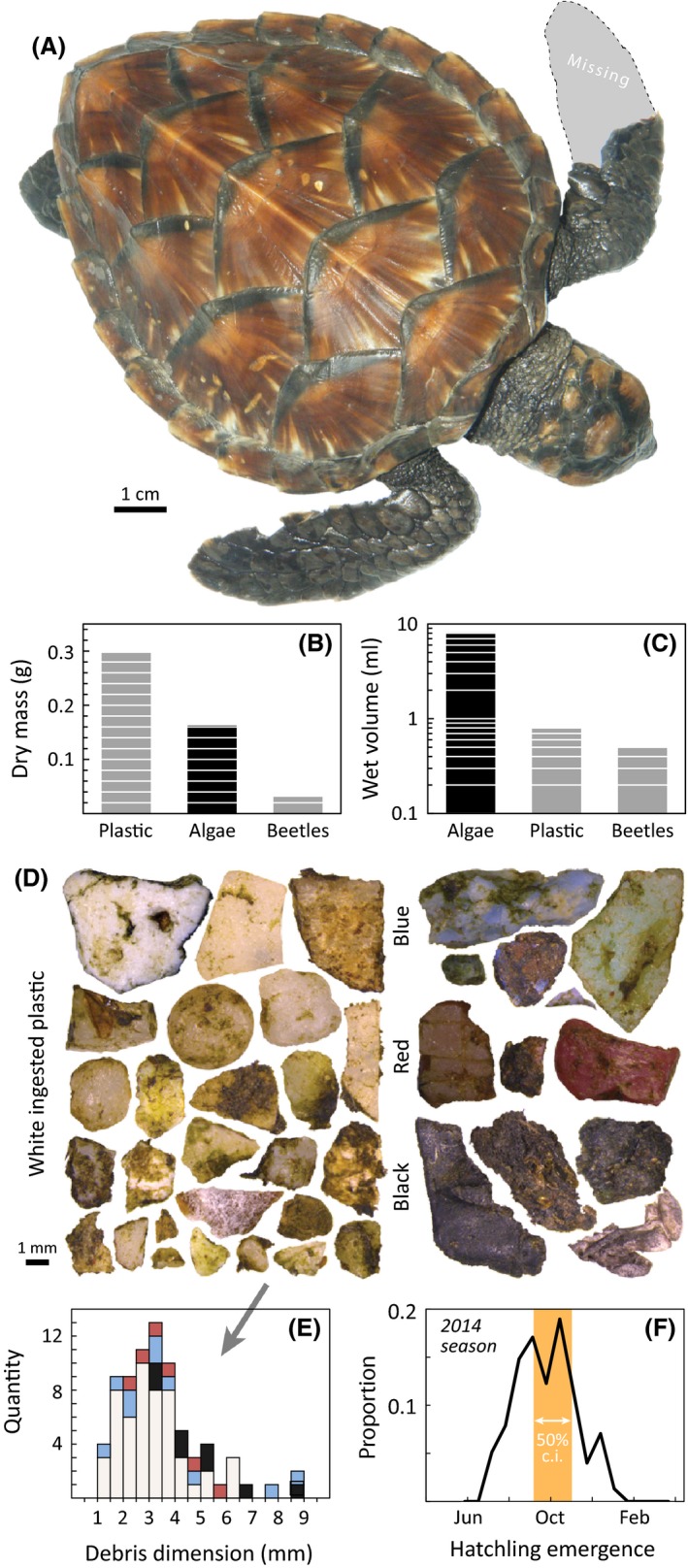
Data obtained from a stranded 9.2 cm hawksbill indicate it resided at the ocean surface, immediately proximate the Main Hawaiian Islands. (A) Image of the turtle stranding from Feb 2015 near Waimea, Kauai (21.968°N, 159.672°W) during a period of exceptionally high surf. Gray area is the silhouetted outline of a presumably depredated left front flipper. Left hind flipper is present but not pictured. (B) Dry mass and (C) wet volume of gastrointestinal tract contents indicate a diet of floating plastic debris, algae, and terrestrial beetles from Kauai Island. White lines are *y*‐axis grid lines. (D) Complete inventory of 41 ingested plastic pieces, (E) tabulated for size‐color frequency each for both length and width. Bar color in (E) corresponds to color labels in (D). (F) 2014 survey records from hawksbill nesting beaches on Maui and Hawaii islands show peak hatchling emergences occurred during Sep–Oct, indicating this turtle was probably 3.4–5.3 months old.

Figure 3 provides details from the 9.2 cm posthatchling that stranded near Waimea, Kauai (21.968°N, 159.672°W) in February 2015 during a period of strong surf. This is the only hawksbill in this cryptic life stage ever examined at necropsy (in 33 years of program operation, 1982–2015), and this presents a novel opportunity. Other than missing ~50% of its left front flipper (Fig. 3A), the turtle was visibly healthy, was given fluids and antibiotics by a veterinarian, yet died within 48 h. The complete gastrointestinal track including the esophagus, stomach, small intestine, and large intestine (Wyneken 2001) measured 57.4 cm. The first 37 cm, comprising all but the large intestine, were empty, while the remaining 20 cm were filled with a mix of pelagic algae, scarab beetles, and plastic debris (Fig. 3B–E). Figure 3B provides the contents by dry mass: 0.30 g plastic (60.5%), 0.16 g algae (33.2%), and 0.03 g beetles (6.3%). Figure 3C provides the wet volume contents: 8.1 mL algae (86.2%), 0.8 mL plastic (8.5%), and 0.5 mL beetles (5.3%). We identified two scarabs as Chinese rose beetles (*Adoretus sinicus*) which are an established invasive agricultural pest in the Hawaiian Islands (McQuate and Jameson 2011), particularly in Kauai. It is likely these beetles blew out onto the ocean from the adjacent agricultural fields in southern Kauai. We counted 41 pieces of plastic debris in the gut: 26 white (dimension ave = 3.1 mm, SD = 1.3), 5 blue (ave = 3.8 mm, SD = 2.5), 4 black (ave = 5.2 mm, SD = 1.3), 3 red (ave = 3.7 mm, SD = 1.2), with three additional fragments of monofilament fishing line (length ave = 16.3 mm, SD = 0.7). We did not trace the microplastic fragments further to their original items of use.

The distribution of emergence dates (Fig. 3F) suggests the posthatchling turtle emerged from its nest sometime in September or October of 2014 placing its age at 4.2 months (range 3.4–5.3 months). As it is most likely this turtle is from the Hawaii population, and given that the majority of hatchlings here are from Pohue beach (55.3%, 3282/5934), this turtle likely travelled at least 500 km during this time, yet was documented in coastal waters. The age, trajectory, and location of this posthatchling turtle are broadly consistent with the data from the drifters we released off Oahu (Fig. 2).

**Figure 4:**
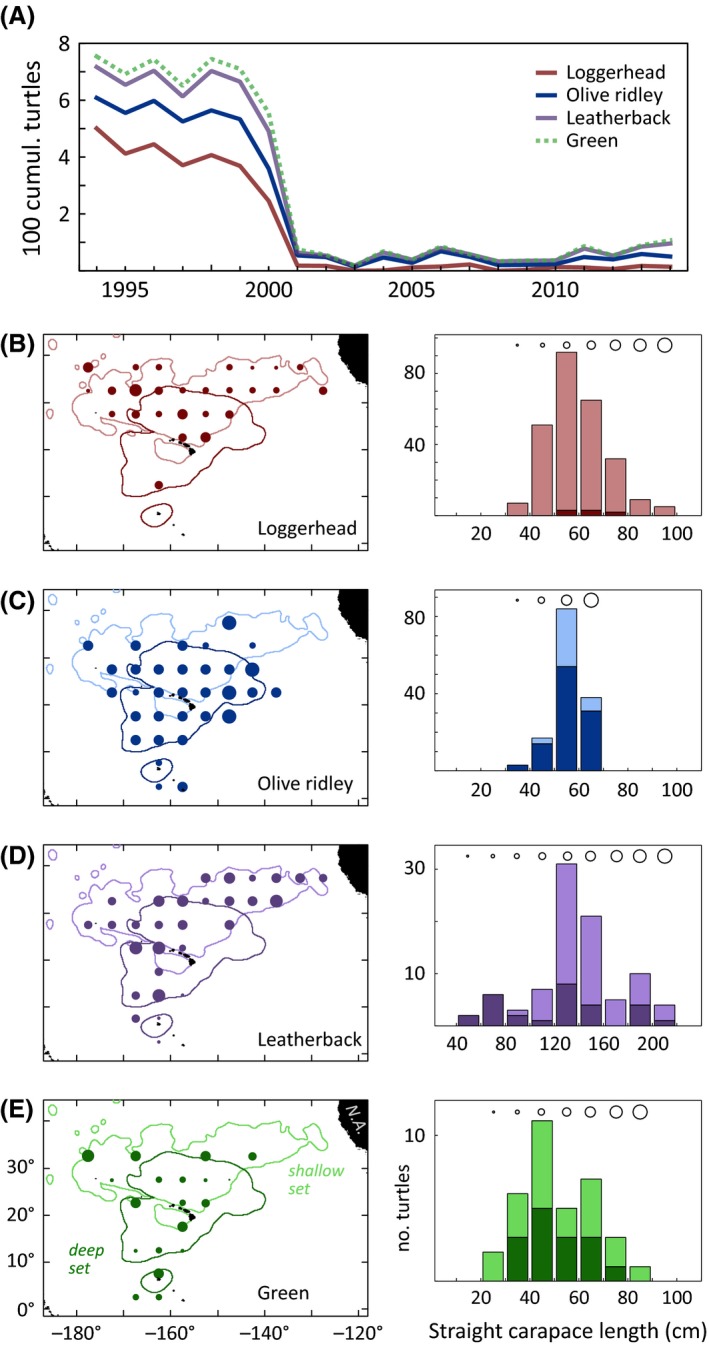
Despite considerable sea turtle bycatch and extensive observer monitoring, the Hawaii‐based longline fleet has recorded zero hawksbill interactions. (A) Observer data from 1994 to 2014 from the tuna‐targeting deep‐set fleet and the swordfish‐targeting shallow‐set fleet suggest roughly 5697 sea turtle interactions. Fishery regulations from 2001 to present have resulted in significantly lower turtle bycatch. Locations and demographics of (B) loggerhead, (C) olive ridley, (D) leatherback, and (E) green turtles caught in fisheries. Light (shallow‐set) and dark (deep‐set) plotted lines are the 95% kernel density estimates of each fishery's extent. Filled circles are bycatch locations from both fisheries, and circle size indicates turtle length corresponding to legend on right panels. Columns in length frequency plots however are colored (as noted above) by fishery. Both fisheries interact with a range of demographics, from young juveniles to breeding adults, from all four species.

Despite considerable fishing effort, Hawaii‐based fisheries have zero documented hawksbill interactions. From 1994 to 2014, the Hawaii‐based longline fisheries put forth a total effort of 329,304 sets containing 638,062,666 hooks. This effort was distributed in 279,930 sets containing 595,706,828 hooks in the deep‐set fleet, and 49,374 sets with 42,355,838 hooks in the shallow‐set fleet. Over this span, we estimate 5697 sea turtles were taken as bycatch, consisting of 2901 loggerheads, 1411 olive ridleys, 1027 leatherbacks, and 358 green turtles. Figure 4a plots the times series of sea turtle bycatch in these fleets, showing the considerable drop in bycatch after fishery management reforms in 2000 (Loggerhead MSRA Technical Advisory Team 2015). From 1994 to 2000, total sea turtle takes averaged 694 year^−1^, but this number was 61 year^−1^ over 2001–2014, a decline of 91.3% in both fleets.

Figure 4B–E plots the spatial distribution and length composition of longline sea turtle bycatch observed. Geographically, the tuna‐targeting deep‐set fishery is positioned somewhat around the MHI, where the swordfish‐targeting shallow‐set effort more closely follows the transition zone chlorophyll front, north of Hawaii (Polovina et al. 2001; Howell et al. 2008). Most observed loggerhead (96.9%, 253/261), leatherback (68.5%, 61/89), and green turtle (57.1%, 20/35) bycatch is in the shallow‐set fishery, where the deep set has the most observed olive ridleys (71.8%, 102/142). Small juveniles from all species, although less common, are taken as bycatch (Fig. 4B–E). Importantly, only juvenile green turtle bycatch is known to originate from Hawaii rookeries (Parker et al. 2011).

## Discussion

Our analysis organized diverse data streams from the North Pacific and Hawaii to understand the cryptic early life history of hawksbill sea turtles. We have several findings of interest. First, hawksbill strandings occur within 1 km of land, except for the 8–34 cm size class that is found up to 10 km offshore (Fig. 1). Second, passive drifters emulating hawksbill and green turtle hatchling dispersal remain in the archipelago for several months or more (Fig. 2). Third, location and stomach contents from the only known posthatchling hawksbill (in this ocean region) indicate the turtle was feeding at the upper ocean surface near land in the first few months of life (Fig. 3). Fourth, despite substantial sea turtle bycatch and a massive effort across space and time, the Hawaii‐based longline fleets have never taken a hawksbill of any life stage, but particularly in the 8–34 cm “lost years” segment (Fig. 4).

Stranding data often serve as an important indicator of population distribution, demographic composition, and threats (Bugoni et al. 2001; Geraci 2005; Chaloupka et al. 2008). Here, we used distance from shore to assess gross habitat preferences throughout development. Only posthatchling to small juveniles (8–34 cm) were observed further than 1 km offshore. The vast majority of all other turtles stranded on shore (75.2%, 85/113) and all within 500 m (Fig. 1). All offshore turtles were entangled in derelict fishing gear, but we do not consider this a bias as nearshore turtles in Hawaii are also often entrapped in fishing gear (e.g., Brunson et al. in review). Additionally, unlike stranding programs in ecosystems with a pronounced continental shelf (e.g., Hart et al. 2006), the Hawaiian Islands are remote oceanic pinnacles and spatial drift is not a major influence to stranding locations (Van Houtan et al. 2010). It is of course possible that hawksbills entangled nearshore could have drifted offshore, but if this was the case, the offshore pattern would be distributed equally among all age classes and not just in the smallest juveniles. This does not happen (Fig. 1), suggesting that 8–34 cm hawksbills may uniquely occupy pelagic waters near the Hawaiian Islands. Bomb radiocarbon aging techniques recently applied to Hawaii hawksbills indicate turtles in this 8–34 cm size class are ≤4 years of age (Van Houtan et al. 2016).

**Figure 5:**
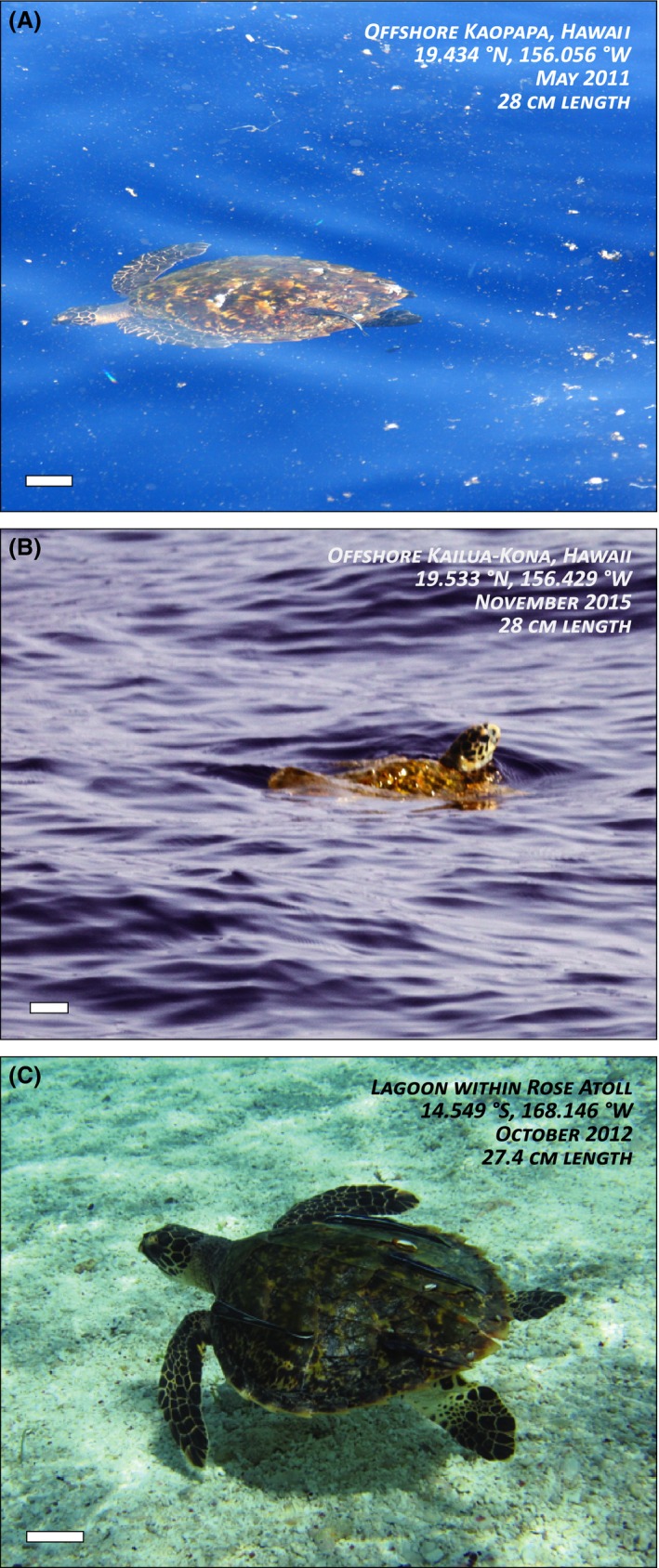
Three in‐water, anecdotal observations of early life‐history hawksbills. Small juvenile hawksbill turtles seen at the ocean surface (A) 13 km off Hawaii Island at 2450 m depth, (B) 47 km off Hawaii Island at 4600 m depth near the Alika Knoll (credit: D. Webster/Cascadia Research). Both turtle lengths estimated at 28 cm SCL. Notice the plentiful debris field floating near the turtle. (C) Small juvenile hawksbill seen foraging in the shallow lagoon of Rose Atoll, a remote oceanic coral pinnacle located 260 km east of the Tutuila Island, American Samoa (credit: K. Van Houtan/NOAA). This turtle was later hand‐captured and measured at 27.4 cm SCL. *Remora* fish, *Pelanes* crabs, and *Lepas* barnacles (only C) were documented on these turtles, which are common pelagic epibionts. Scale bar in all images is 5 cm.

Beyond stranding observations, two 28 cm juvenile hawksbills have recently been documented off the Kona (west) coast of Hawaii Island. Both turtles were seen swimming freely at the ocean surface during a regular coastal cetacean survey by the Cascadia Research Collective (e.g., Baird et al. 2013; also see Appendix S1). One turtle was observed in May 2011 for <1 min, 13 km offshore, in waters 2450 m deep (Fig. 5A). A second turtle was observed in November 2015 for about the same duration, but 47 km offshore, at 4600 m depth, and near an oceanographic feature known as the Alika Knoll (Fig. 5B). Outside of the strandings, these are the only such records of juvenile hawksbills offshore in Hawaii. Additionally, three small juvenile hawksbills (35–46 cm) have been recently recorded in nearshore reefs of remote NWHI atolls (Van Houtan et al. 2012) and juvenile hawksbills in this cryptic life‐history stage have been observed with some frequency in the shallow lagoon of Rose Atoll, American Samoa (Fig. 5B, Pfaller et al. 2014). Of the latter group, one turtle was captured and measured at 27.4 cm SCL and was one of numerous small juvenile hawksbills seen in the Rose Atoll lagoon.

There is a growing appreciation for how physical oceanography shapes the early life history of sea turtles (Hays et al. 2010; Monzón‐Argüello et al. 2012; Putman et al. 2014; Ascani et al. in revision). Although our drifter tag life and data are limited, trajectories from both species' nesting grounds show variable movements for several months with longer lived tags drifting zonally beyond 180° (Fig. 2). This pattern suggests hawksbill and green hatchlings from Hawaii could mature at sea in oceanic ecosystems or perhaps even at neritic foraging areas in the distant western Pacific. Recent genetics studies on Hawaii green turtles indicate the population is isolated to the Central Pacific (Dutton et al. 2008; Seminoff et al. 2014). Published hawksbill migrations show that 11 of 13 females nesting in Maui and Hawaii islands return to nearby foraging grounds (Graham 2009; Parker et al. 2009, 2014). For the two other tracked hawksbills, one stopped transmitting west of Kauai (Parker et al. 2014), and the other was last observed at 14.731°N, 175.106°W (Graham 2009), but these studies suggest turtle mortality may have preceded the track's westward drift. The latter location is roughly 250 km WSW of Johnston Atoll, just 128 km south of the track from our longest observed hawksbill drifter (Fig. 2). Although drifter data should be considered within the context of swimming behavior (Putman and Mansfield 2015), more genetics research, satellite tracking, and dispersal modeling may help resolve the developmental biogeography of hawksbills. Currently, no empirical data exist on the innate navigational swimming for hawksbills (Putman et al. 2014) for any population.

Although the posthatchling examined in this study represents one single individual, it is the only recorded specimen of this cryptic demographic stage for the population and therefore of significance. Our estimates of age and natal migration distance place the turtle at 4 months old (Fig. 3F) having travelled approximately 500 km, consistent with our MHI drifters (Fig. 2). The collection of floating dietary items indicates the turtle was feeding at the upper ocean surface (perhaps restricted to the neustal zone) proximate to a landmass. Terrestrial insects have not been previously recorded as dietary items in Hawaiian sea turtles. The presence of two *A. sinicus* scarabs in the intestine suggests the turtle was feeding close to Kauai Island where this beetle is a dominant agricultural pest (McQuate and Jameson 2011).

Microplastics were the largest dietary item by mass (Fig. 3B), representing 41 pieces all <1 cm (Fig. 3D–E). Such plastics are a growing concern for marine life (Bugoni et al. 2001; Lazar and Gračan 2011) as they are a known vector for organic pollutants and pose an energetic opportunity cost (Santos et al. 2015). Our results complement those of a recent study that found marine debris ingestion a serious threat for oceanic, bycaught sea turtles in the North Pacific (Wedemeyer‐Strombel et al. 2015), but did not analyze hawksbills (as they are not present in bycatch). If the plastics recovered from the posthatchling intestine are representative of the diet of this and other such hawksbills, this may contribute to delayed development, and even mortality, at the population level. While this does not affect our aging of this turtle, which is based on emergence data, it suggests turtles of this age that avoid plastics might be larger. Confirming the threat of debris ingestion, one of the juvenile hawksbills documented off the Kona Coast was observed in a convergence front, amidst significant floating debris (Fig. 5A). Future research therefore might examine the origins and impacts of microplastics in pelagic Hawaiian sea turtles.

With 20 years of operation, spanning 50° longitude and 35° latitude, over half a billion hooks deployed, and with nearly 6000 bycaught sea turtles (Fig. 4A), it is remarkable that no hawksbills have been taken in the Hawaii‐based longline fisheries. This lack of hawksbill bycatch in Pacific ocean longlines, and scant presence in Atlantic longlines, has been observed previously (Lewison et al. 2004; Finkbeiner et al. 2011). The simple interpretation of these fishery‐dependent data is that Hawaii hawksbills do not occupy such oceanic habitats, but are restricted to neritic and pelagic waters proximate to landmasses, similar to other populations (e.g., Gaos et al. 2012b). Our other data streams concur. The lack of hawksbill bycatch could also be an artifact of its small population size, however.

Hawaii hawksbills are probably the smallest sea turtle population on the planet. The population is significantly smaller than the Hawaiian green turtle population caught in these longline fisheries (Seminoff et al. 2014), smaller than other sea turtle populations also caught in these longline fisheries, and smaller than other hawksbill populations from other geographic regions (Hutchinson et al. 2008). Considering the sheer magnitude of longline effort, and the observed numbers of turtles caught by the fleets (Fig. 4A), if hawksbills reside in the oceanic ecosystem in the North Pacific, however, we might expect low levels of longline interactions. We formalize this by calculating the expected number of hawksbill interactions in the Hawaii‐based longline fleet, based on population size alone. We do this considering empirical data on population productivity, known recruitment sizes, size‐at‐age models, stage‐specific annual survival, and inferences on gear selectivity – all by comparison to green turtles (see Supplemental Online Material). Based on the population abundance alone, this routine suggests 1.2–3.1 hawksbill interactions are expected, but that given gear selectivity or catchability (e.g., Millar and Methot 2002), the estimates might increase to 2.0–5.0 hawksbills. As we have observed zero hawksbill interactions, we may conclude that differences in population size, or perhaps even gear selectivity, are perhaps not a contributing factor to the absence of hawksbill bycatch. Other factors, potentially such as habitat choice, foraging habits, or neonate navigation, might be significant in the observed patterns. However, we urge interpretive caution, as our estimated bycatch extremes of 1–5 hawksbills expected are near the observation of zero.

Figure 4C and D shows the deep‐set fleet uniquely catches the smallest demographics of olive ridleys and leatherbacks – both species known to have pronounced oceanic phases. For leatherbacks, in particular, the smallest juveniles are bycaught toward the equator (Fig. 4D) consistent with proposed physiological restrictions (Jones et al. 2012). The smallest demographics of loggerhead and green turtles are caught closer to the ocean surface in the shallow‐set fleet (Fig. 4B and E). The region spanning 30°–40°N constitutes a particularly critical region in the early development of loggerheads (Ascani et al. in revision). The most common fisheries reporting hawksbills, globally, are commercial and artisanal fisheries that operate in coastal waters and close to the ocean surface (Finkbeiner et al. 2011; Liles et al. 2011). Besides U.S. fisheries in the North Pacific, observers in the Japanese high seas driftnet fishery reported catching one 43 cm hawksbill at 29.000°N, 173.000°E (Wetherall et al. 1993) in the early 1990s – but many details surrounding this data point are unspecified. Such driftnet fisheries operate at the ocean surface above depths typical of longline gears and likely have higher interaction rates with all species of small juvenile turtles. However, driftnet fleets are no longer in operation (Wetherall et al. 1993), and to our knowledge, hawksbills have never been recorded in any Pacific longline fisheries.

Similar to our findings here, data from Indonesian fisheries indicate early developmental hawksbills reside in coastal pelagic ecosystems. The magnitude of historical hawksbill take in the coastal waters of western Indo‐Pacific, however, dwarfs the incidental bycatch from Hawaii‐based commercial fishing vessels (even with all species combined). Market surveys from the early 1970s estimate 40,000 juvenile hawksbills were taken annually by surface spear fishermen in the coastal waters off Sumatra and Sulawesi (Kajihara and Uchida 1974; Balazs and Nozoe 1978). These turtles ranged in size from 18 to 35 cm, which the studies estimated at 1–2 years of age, and were captured in pelagic waters <50 km from land. These results corroborate ours from the Hawaiian archipelago, especially from anecdotal observations (Van Houtan et al. 2012), stranding data (Fig. 1), and in‐water surveys (Fig. 5).

Our effort here is to understand the early life history of hawksbill sea turtles using diverse data streams. Previously, we applied this approach to the historical ecology of sea turtles in Hawaii (Van Houtan et al. 2012; Van Houtan and Kittinger 2014). Our analysis of strandings, stomach contents, satellite drifters, and longline bycatch records spans several decades and covers a large portion of the North Pacific. While no single individual data stream may alone have definitive statistical power, this is perhaps largely due to the inescapable situation that Hawaii hawksbills are an extremely small population, and as a result our data are few. However, despite these deficiencies, the different data streams paint a similar picture, and collectively provide novel and potentially important insights. Further progress on the spatial population structure of hawksbills in the Pacific may be understood more directly from satellite tracking of hatch‐year turtles, and from isotopic analysis of tissues (Reich et al. 2007). Our observations document that Hawaii hawksbills spend the first 4 years of their development in coastal pelagic waters and in neritic habitats of remote atolls. The oceanographic dynamics and threats in coastal areas are then critical for understanding the conservation status for hawksbills in Hawaii, and perhaps beyond. Although hawksbills have not been observed by longline fisheries in the oceanic habitats of the North Pacific, we cannot rule out the possibility that they may reside there and that threats in those regions as well may factor in their long‐term population persistence.

## Conflict of Interest

None declared.

## Supporting information


**Appendix S1.** Estimating expected hawksbill fishery interactions based on population productivity.
**Table S1.** Calculating the expected number of hawksbill interactions in the Hawaii‐based longline fisheries based on population numbers.
**Figure S1.** Map survey tracklines performed by the Cascadia Research Collective CRC) from January 2000 through January 2016.
