# Supplementary material for: The developmental biogeography of hawksbill sea turtles in the North Pacific
Source: Ecol Evol. 2016 Mar 8;6(8):2378–89. doi: 10.1002/ece3.2034 (PMC4834323; doi:10.1002/ece3.2034)
Supplement: Supplementary file 1 — Appendix S1. Estimating expected hawksbill fishery interactions based on population productivity. Table S1. Calculating the expected number of hawksbill interactions in the Hawaii‐based longline fisheries based on population numbers. Figure S1. Map survey tracklines performed by the Cascadia Research Collective CRC) from January 2000 through January 2016. [file ECE3-6-2378-s001.docx]

**SUPPLEMENTAL ONLINE MATERIAL FOR:**

***THE DEVELOPMENTAL BIOGEOGRAPHY OF HAWKSBILL SEA TURTLES***

***IN THE NORTH PACIFIC***

**BY VAN HOUTAN, FRANCKE, ALESSI, *ET AL*.**

**SUBMITTED TO ECOL. EVOL.**

**Estimating Expected Hawksbill Fishery Interactions Based on Population Productivity**

The purpose of this section is to develop a quantitative framework for determining the expected hawksbill sea turtle interactions in the Hawaii-based longline fisheries. To accomplish this, we rely on: (i) empirical data on population productivity, (ii) published size-at-age models from von Bertalanffy growth functions (VBGF), (iii) published values of stage specific annual survival, and (iv) observed sea turtle bycatch in the Hawaii-based longline fisheries. These data provide us a means to calculate the total abundance of the at-sea sea turtle population in any given year.

For the annual population productivity of Hawaiian green turtles, we rely on the data contained in the recent global status review for the species ([Seminoff et al. 2014](#_ENREF_9)) and a forthcoming review of the demographic features of the population ([Balazs et al. in review](#_ENREF_2)). Accordingly, we expect 951 nesters yr^-1^, and each nester to lay on average 2.9 nests, resulting in 2,729 nests yr^-1^. Each nest contains 92.4 eggs and has an emergence rate of 71% resulting in 179,058 hatchlings yr^-1^. Given that green turtles recruit to the Main Hawaiian Islands at approximately 35 cm straight carapace length (SCL, see: [Chaloupka et al. 2008](#_ENREF_4), [Van Houtan et al. 2010](#_ENREF_13)), we need to understand the age of turtles at this recruitment size. Using the VBGF model with the parameters *t*_0_ = -0.181, *L*_oo_ = 89.7, and *k* = 0.173 ([Van Houtan 2015](#_ENREF_10), [Balazs et al. in review](#_ENREF_2)), a Hawaiian green turtle 35 cm in length is 2.7 years of age. Assuming constant population production using the above data, we assume annual survival of hatch year turtles is 0.35 and in subsequent years for pelagic turtles is 0.80 ([Van Houtan et al. 2014](#_ENREF_12)). Combining these data, we add the abundance of the turtles in the cohorts that are age 1, 2, and 2.7 years to determine the total abundance of the “lost years” cohort of Hawaiian green turtles available for bycatch by the longline fleets. Considering the sizes of turtles caught by these fleets, we exclude hatch year turtles from this group ([see main text and Parker et al. 2011](#_ENREF_6)). From these calculations, the total number expected in this oceanic phase is 143,157 turtles. Table S1 presents a full summary of these data and the published studies from which we obtain parameters and variables.

For the annual population production of Hawaiian hawksbill sea turtles, we rely on the comprehensive project report that reviewed data and results from the first 20 years of population monitoring ([Seitz et al. 2012](#_ENREF_8)). However, considering that monitoring effort on this project was inconsistent through time and space, we have three scenarios for annual nesting activity. We consider a low, median, and high estimate to be 15, 20, and 25 nesters yr^-1^ in the population to account for this variable activity, which accounts for currently undocumented nests on Maui, Molokai, and Kauai that contribute to the population production ([Seitz et al. 2012](#_ENREF_8)). With this in mind, females lay on average 3.3 nests yr^-1^, resulting in a range of 50-83 nests yr^-1^ expected. Each nest contains 175.2 eggs and has an emergence rate of 71.9% resulting in 6,235-10,392 hatchlings yr^-1^. Given that hawksbill turtles recruit to the Main Hawaiian Islands at approximately 35 cm straight carapace length (see Figure 1, main text) we need to understand the age of turtles at this recruitment size. Using the VBGF model with the parameters *t*_0_ = -0.171, *L*_oo_ = 81.0, and a low, median, and high *k* values of 0.098, 0.128, and 0.21 ([Van Houtan et al. 2016](#_ENREF_11)), a Hawaiian hawksbill turtle 35 cm in length is 2.5-5.6 years of age. Assuming constant population production using the above data, we constrained annual survival of hatch year turtles to 0.35, and other juvenile pelagic turtles to 0.80 as above. Combining these data, we add the abundance of the turtles in the annual cohorts as above for the low, median, and high scenarios to determine the range of total abundance of the “lost years” cohort, again excluding hatch-year turtles. From these calculations, the total number expected in this oceanic phase is 5,482-13,530 turtles.

Now that we have estimates of the at-sea life stage (i.e. “lost years” turtles) abundance, we can compare the ratio of these two populations to the observed interactions of Hawaiian green turtles to infer what we might expect hawksbill interactions, given population abundance alone. Due to partial observer coverage, we estimated a total of 358 green turtle interactions in the longline fisheries during the total period (see main text Figure 4), but only observed 54 green turtle takes. Of these 54 green turtle takes, we expect only 32 to be from the Hawaii population. We determined this from the number of green turtles taken meridionally above 20°N, as a result of genetic analyses presented in [Parker et al. (2011](#_ENREF_6)). Considering the range of abundance scenarios, the ratio of “lost years” greens: “lost years” hawksbills is 10.6, 14.3, and 26.1. If we apply these ratios to the 32 observed Hawaiian green turtles taken in these fleets, we then might expect between 1.2-3.1 hawksbills taken at sea by these longline fisheries, just considering population abundance with all other things being equal.

Furthermore, we consider the observed frequency of fisheries interactions in nearshore strandings in the Main Hawaiian Islands for each species. We do this perhaps as a crude measure of foraging habits that may affect gear selectivity ([e.g., Millar and Methot 2002](#_ENREF_5)). For hawksbills, 54.2% of strandings had fishing gear interactions ([Brunson et al. in review](#_ENREF_3)), where only 33.2% of green turtles had fishing gear interactions ([unpubl. data, but also see Chaloupka et al. 2008](#_ENREF_4)). If we correct the above number of expected hawksbill interactions for this seeming preference for fishing gear (multiply by 0.533/0.187), the expected number of hawksbill interactions could be as high as 2.0-5.0 turtles. Again, Table S1 presents a full summary of these data and the published studies from which we obtain parameters and variables.

Considering that zero hawksbills were actually observed taken in these fleets during this time, we can conclude that differences in population size, or perhaps even gear selectivity, are likely not a contributing factor to the absence of hawksbill bycatch. Therefore other factors, potentially such as habitat choice or innate navigation ([Putman and Mansfield 2015](#_ENREF_7)), may contribute to the observed patterns.

| **POPULATION VARIABLE** | **TURTLE ABUNDANCE** | | | **REFERENCE** |
| --- | --- | --- | --- | --- |
|  | **LO** | **MED** | **HI** |  |
|  |  |  |  |  |
| ***Hawaiian green turtles*** |  |  |  |  |
| nesters yr-1 | -- | 951 | -- | Seminoff *et al.* 2014 |
| clutches nester-1 | -- | 2.9 | -- | Balazs *et al.* (2015) |
| nests yr-1 | -- | 2,729 | -- | Seminoff *et al.* 2014 |
| eggs yr-1 | -- | 252,194 | -- | Seminoff *et al.* 2014 |
| hatchlings yr-1 | -- | 179,058 | -- | Seminoff *et al.* 2014 |
| VBGF *t0* | -- | -0.181 | -- | Balazs *et al.* (2015) |
| VBGF *Loo* | -- | 89.7 | -- | Balazs *et al.* (2015) |
| VBGF *k* | -- | 0.173 | -- | Van Houtan 2015 |
| recruitment size (SCL) | -- | 35.0 | -- | PIFSC data |
| recruitment age (years) | -- | 2.7 | -- | this study |
| annual survival, hatch year | -- | 0.35 | -- | Van Houtan *et al.* (2014) |
| annual survival, pelagic juvenile | -- | 0.80 | -- | Van Houtan *et al.* (2014) |
| "Lost years" abundance (turtles) | -- | **143,157** | -- | this study |
| Interactions\| corrected, total | -- | 358 | -- | this study |
| Interactions\| observed, total | -- | 54 | -- | this study |
| Interactions\| observed, Hawaiian pop. | -- | 32 | -- | Parker *et al.* 2012 |
|  |  |  |  |  |
| ***Hawaiian hawksbill turtles*** |  |  |  |  |
| nesters yr-1 | 15 | 20 | 25 | Seitz *et al.* 2012, this study |
| clutches nester-1 | 3.3 | 3.3 | 3.3 | Seitz *et al.* 2012 |
| nests yr-1 | 50 | 66 | 83 | Seitz *et al.* 2012 |
| eggs yr-1 | 8,672 | 11,563 | 14,454 | Seitz *et al.* 2012 |
| hatchlings yr-1 | 6,235 | 8,314 | 10,392 | Seitz *et al.* 2012 |
| VBGF *t0* | -0.171 | -0.171 | -0.171 | Van Houtan *et al.* (2016) |
| VBGF *Loo* | 81 | 81 | 81 | Van Houtan *et al.* (2016) |
| VBGF *k* | 0.21 | 0.128 | 0.098 | Van Houtan *et al.* (2016) |
| recruitment size (SCL) | 35.0 | 35.0 | 35.0 | PIFSC data |
| recruitment age (years) | 2.52 | 4.25 | 5.60 | this study |
| "Lost years" abundance (turtles) | **5,482** | **9,999** | **13,530** | this study |
| ratio LY greens: LY hawksbills | 26.1 | 14.3 | 10.6 | this study |
| Interactions\| observed, total | 0 | 0 | 0 | this study |
| Interactions\| observed, Hawaiian pop. | 0 | 0 | 0 | this study |
| interactions\| expected, observed | **1.2** | **2.3** | **3.1** | this study |
| interactions\| expected, gear selectivity | 2.0 | 3.7 | 5.0 | this study |

**Table S1. Calculating the expected number of hawksbill interactions in the Hawaii-based longline fisheries based on population numbers.** This table provides the details from the calculations described in the Supplement Online Material. All variables and parameters are described in the main text or in the listed published studies. The calculations are limited to green and hawksbill sea turtles originating from rookeries in Hawaii, and then observed in takes by the Hawaii-based longline fisheries which operate over a large swath of the North Pacific Ocean (see main text Figure 4). “Lo”, “Med”, and “Hi” abundance scenarios are for the low, median, and high nesting scenarios described in the Supplement.

**
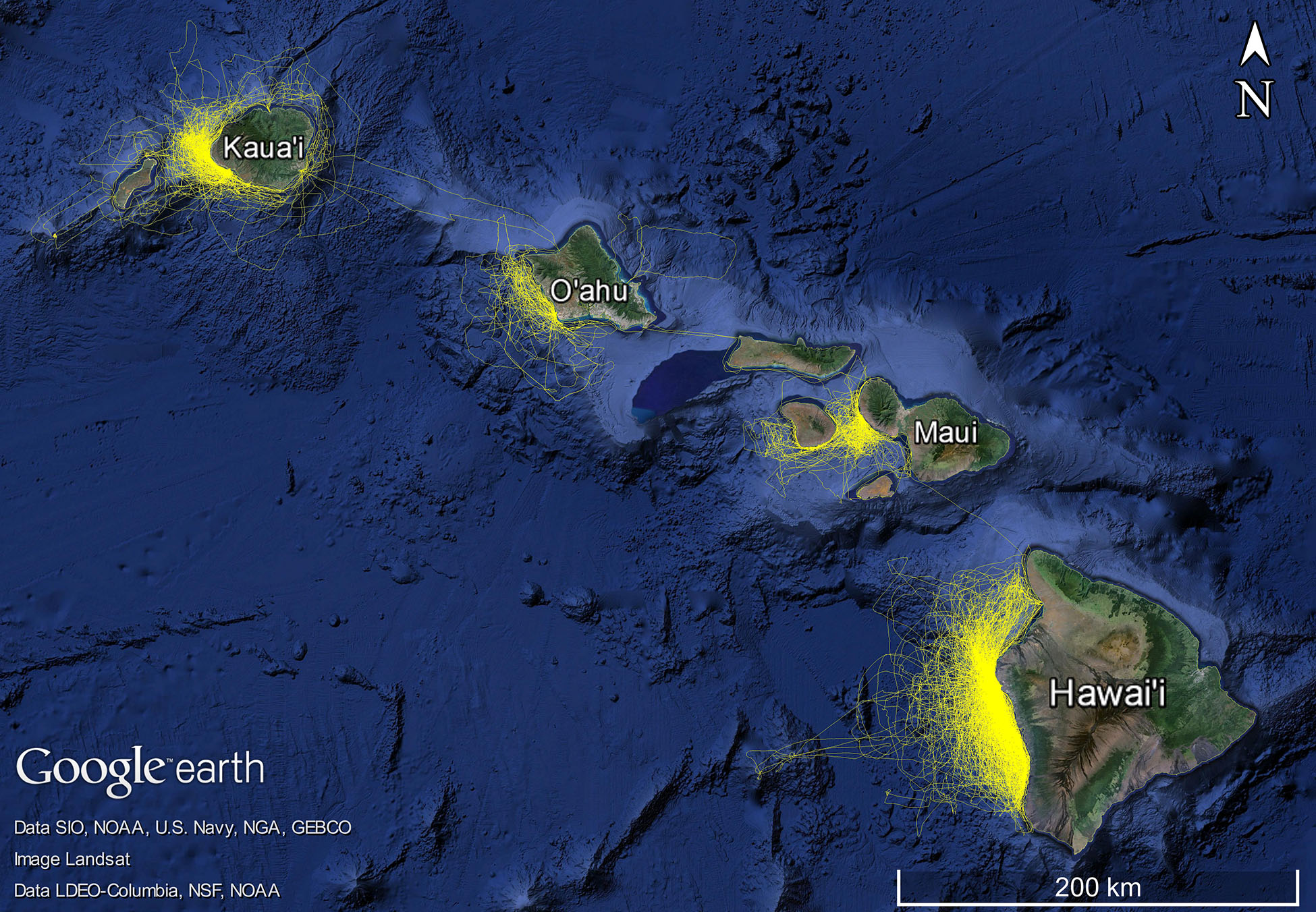
**

**Figure S1. Map survey tracklines performed by the Cascadia Research Collective CRC) from January 2000 through January 2016.** Nearly 109,000 km of trackline of small vessel surveys were conducted over a 13-year period in the coastal pelagic waters of the Main Hawaiian Islands. The aim of these surveys is primarily to record cetacean observations, with additional notable marine life recorded. During these surveys two juvenile hawksbills (each measuring 28 cm +/- 3 cm length) were documented, both in the waters off the west coast of the island of Hawaii (See Figure 5 from the main text). On both occasions, CRC observers were < 15 m distance to the turtles. Further details on the survey methods and cetacean observations are provided by [Baird et al. (2013](#_ENREF_1)).

**ACCESS TO DATA USED IN THIS STUDY**

The data used in this study are all under the domain of the United States federal government; here with the National Oceanic and Atmospheric Agency (NOAA), National Marine Fisheries Service (NMFS). As a result, these data are freely accessible through agency web servers. The primary interface for access of these data is through the NMFS Enterprise Data Management Program, InPort (Release 3.4.1.1, <https://inport.nmfs.noaa.gov/inport>). This public interface is relatively recent, and not unexpectedly, new agency data streams are continuously being added.

For direct program contact, support, and metadata interpretation, there are different policies and points of contact for the different data streams used in the study. All data on fishery operator locations (and therefore, indirectly, bycatch event coordinates) are subject to confidentiality agreements under the Magnuson-Stevens Fishery Conservation and Management Reauthorization Act. As a result such information is only released after the requestor signs appropriate access and use agreements. The data are still open access, however, after such end user agreements are made.

The Pacific Islands Regional Office (PIRO) observer program - who maintain protected species bycatch records - are reachable at <http://www.fpir.noaa.gov/OBS/obs_observer_database.html>. The Pacific Islands Fisheries Science Center (PIFSC), Fisheries Resource and Monitoring Division can be reached at <http://www.pifsc.noaa.gov/frmd/mail.php>, and they maintain the fishery operator and catch data. The PIFSC Protected Species Division operates the sea turtle stranding and response program and they can be reached at <http://www.pifsc.noaa.gov/psd/mail.php>.

All these data and contact links will provide direct access to the data used in this study.

**REFERENCES**

Baird, R. W., D. L. Webster, J. M. Aschettino, G. S. Schorr, and D. J. McSweeney. 2013. Odontocete Cetaceans Around the Main Hawaiian Islands: Habitat Use and Relative Abundance from Small-Boat Sighting Surveys. Aquat. Mamm. 39:253-269.

Balazs, G. H., K. S. Van Houtan, S. K. Hagrove, S. Brunson, and S. K. K. Murakawa. 2015. A review of the demographic features of Hawaiian green turtles (*Chelonia mydas*). Chel. Conserv. Biol. 14(2):119-129.

Brunson, S., S. K. Hagrove, K. S. Van Houtan, G. H. Balazs, and T. M. Work. in review. Three decades of hawksbill sea turtle strandings in the Hawaiian Islands.

Chaloupka, M., T. M. Work, G. H. Balazs, S. K. K. Murakawa, and R. Morris. 2008. Cause-specific temporal and spatial trends in green sea turtle strandings in the Hawaiian Archipelago. Mar. Biol. 154:887-898.

Millar, R. B. and R. D. Methot. 2002. Age-structured meta-analysis of US West Coast rockfish (Scorpaenidae) populations and hierarchical modeling of trawl survey catchabilities. Can. J. Fish. Aquat. Sci. 59:383-392.

Parker, D. M., P. H. Dutton, and G. H. Balazs. 2011. Oceanic diet and distribution of haplotypes for the green turtle, Chelonia mydas, in the Central North Pacific. Pac. Sci. 65:419-431.

Putman, N. F. and K. L. Mansfield. 2015. Direct Evidence of Swimming Demonstrates Active Dispersal in the Sea Turtle “Lost Years”. Curr. Biol.

Seitz, W. A., K. M. Kagimoto, B. Luehrs, and L. Katahira. 2012. Twenty years of conservation and research findings of the Hawai‘i Island Hawksbill Turtle Recovery Project, 1989 to 2009. Pacific Cooperative Studies Unit Technical Report, University of Hawaii-Manoa, Department of Botany, Honolulu, HI.

Seminoff, J. A., G. H. Balazs, P. H. Dutton, T. Eguchi, H. L. Haas, S. A. Hargrove, D. L. Klemm, A. M. Lauritsen, S. L. MacPherson, P. Opay, E. E. Possardt, K. S. Van Houtan, and R. S. Waples. 2014. Green turtle (*Chelonia mydas*) Status Review under the U. S. Endangered Species Act. NOAA National Marine Fisheries Service, Silver Spring, MD.

Van Houtan, K. S. 2015. Impacts of incidental bycatch from the American Samoa-based longline fishery to marine turtle populations, Internal Report IR-15-027. NOAA Fisheries, Pacific Islands Science Center, Honolulu, HI USA.

Van Houtan, K. S., A. H. Andrews, T. T. Jones, S. K. K. Murakawa, and M. E. Hagemann. 2016. Time in tortoiseshell: a bomb radiocarbon-validated chronology in sea turtle scutes. Proc. R. Soc. B. 283:20152220.

Van Houtan, K. S., S. Hargrove, and G. H. Balazs. 2014. Modeling sea turtle maturity age from partial life history records. Pac. Sci. 68:465-477.

Van Houtan, K. S., S. K. Hargrove, and G. H. Balazs. 2010. Land Use, Macroalgae, and a Tumor-Forming Disease in Marine Turtles. PLoS ONE 5:e12900.
